# The early efficacy of Heller myotomy in the treatment of Iranian patients with achalasia 

**Published:** 2016

**Authors:** Saeed Abdi, Mojgan Forotan, Abdolrahim Nikzamir, Saeedeh Zomorody, Somayeh Jahani-Sherafat

**Affiliations:** 1 *Gastroenterology and Liver Diseases Research Center, Research Institute for Gastroenterology and Liver Diseases, Shahid Beheshti University of Medical Sciences, Tehran, Iran*; 2 *Biochemical Department, Faculty of Medicine, Shahid Beheshti University of Medical Sciences, Tehran, Iran*

**Keywords:** Achalasia, Heller myotomy, Manometry

## Abstract

**Aim:**

The purpose of this study was to determine the efficacy of Heller myotomy for the treatment of achalasia in a referral center in Tehran, and investigate the clinical characteristics, manometric results and treatment responses among three achalasia subtypes in Iranian patients.

**Background:**

Esophageal achalasia is an unusual swallowing disorder, characterized by high pressure in the lower esophageal sphincter (LES) on swallowing, failure relaxation of the LES and the absence of peristalsis in esophageal.

**Patients and methods:**

In this cross sectional study, clinical symptom and esophageal manometry before and 2 months after treating with Heller myotomy in 20 patients with achalasia who were referred to Taleghani Hospital, Tehran, in 2013 were evaluated. Patients’ demographic, clinical features and response to treatment were analyzed using SPSS software (version 20, Chicago, IL, USA).

**Results:**

All the diagnostic criteria measured after the treatment were significantly different (P<0.05) before and after the therapy. The average decline in the length of the esophagus was 1.8 cm and dysphasia score was 7.25 units. Also an average decline in LES Resting Pressure, LES Residual Pressure, PIP, and IRP were 23.2 mmHg, 14.3 mmHg, 3.4 mmHg and 17.8 mmHg, respectively.

**Conclusion:**

Results of this study showed that the Heller myotomy is highly effective in relieving dysphasia in patients with achalasia. Also, type II achalasia is the most common subtype of achalasia with a better response to Heller myotomy compared to the other types.

## Introduction

 Esophageal achalasia is an unusual swallowing disorder relating the smooth muscle layer of the esophagus and the lower esophageal sphincter (LES) that affects about 1 in every 100,000 people and most subjects are diagnosed between 25 and 60 years old (1-4).

 Major symptoms of achalasia in more than 80% of patients are usually difficulty with swallowing, regurgitation, dysphasia, and chest pain (1, 5). These symptoms are due to the absence of peristalsis in esophageal, failure relaxation LES and high pressure in the lower esophageal sphincter (LES) on swallowing (6).

Achalasia is due to the disintegration of neurons in the esophageal wall. Regarding the histological examination, previous reports have revealed that reduced quantities of ganglion cells in the myenteric plexuses, and the ganglion cells that remain regularly are bounded by lymphocytes and eosinophils (7, 8). In the preliminary phase of the achalasia (vigorous achalasia), disintegration of inhibitory nerve fibers in the esophagus leads to unobstructed action of excitatory neurotransmitter, and high amplitude non-peristaltic. However, in the late phase of the achalasia (classic achalasia), progressive loss of cholinergic neurons results in dilation and low amplitude simultaneous contractions in the esophageal body (9).

Several options are offered for the treatment of achalasia, but none can reverse the injury of nerve cells in the esophagus. However, the treatments are usually effective for improving the symptoms. The standard treatment of esophageal achalasia focuses on the LES degradation to decrease sphincter pressure. LES degradation can be achieved by pneumatic dilatation, Heller myotomy, botulinum toxin injection, pharmacotherapy and peroral endoscopic myotomy (POEM) (10). 

Pharmacotherapy is usually unsuccessful. Also, pneumatic dilation with balloons are usually less successful in young patients. Therefore, Heller myotomy in patients wishes not to accept the 3% to 5% puncture risk is becoming gradually more popular for treatment of achalasia (11, 12) and this satisfactory might be due to less postoperative pain, fewer parietal complications and shorter hospital stay, and is now considered as the first line treatment (13). 

A robotic assisted laparoscopic Heller myotomy (LHM), as a substitute approach was first reported in 2001 by Melvin et al. (14). Short-term follow-up studies have verified similar results to those of LHM. In a comprehensive study on 149 patients who underwent Heller myotomy, Ortiz et al. showed satisfactory results in 90 % of patients after 5 years (15).

Recent studies classified the achalasia into three subtypes including, type I, II and III. According to the new achalasia classification, each subtype has diverse clinical and manometric features and type II is the most common subtype. Moreover, type II and type III are associated with good and poor treatment response, respectively (16-18). However, there is no clinical record regarding the achalasia subtypes in the Iranian population. Therefore, the purpose of this study was to determine the early efficacy of Heller myotomy for the treatment of achalasia in a referral center in Tehran, Iran. Another aim of this study was to investigate clinical characteristics, manometric results and treatment responses among three achalasia subtypes in Iranian patients. 

## Patients and Methods

**Patients**

Twenty patients who were diagnosed with achalasia underwent a Heller myotomy at GI ward of Taleghani Hospital (referral center of gastrointestinal disorders in Tehran, Iran) during 2013. Diagnosis of achalasia was definite by manometry and based on the absence of peristalsis in esophageal, failure relaxation of the LES and high pressure in the LES on swallowing (Inclusion criteria). Exclusion criteria included: pregnancy, patients older than 50 years old, achalasia associated with esophageal or gastric carcinoma and subject with prior therapy.

After giving informed written consent, collected patients were completed a standardized pretreatment measurement consisting of esophageal manometry, clinical symptom evaluation and assigned to undergo Heller myotomy. Demographic characteristic such as age, gender, and clinical features including dysphasia score, chest pain and regurgitation as well as manometrical parameters including LES resting pressure, LES residual pressure, free drinking esophagus, PIP, IRP, and length of the esophagus were completed for each patient by a valid questionnaire. To determine improvement in symptoms, patients were evaluated two months after myotomy and underwent an additional manometry. 

According to the Chicago classification, achalasia is classified into 3 types. Type I (classical achalasia; no evidence of pressurization), type II (achalasia with compression or compartmentalization in the distal esophagus >30 mm Hg) and type III (vigorous achalasia or two or more spastic contractions). 

The total symptom scores consisting of dysphagia to solid and liquids, active and passive regurgitation and chest pain, were recorded. The frequency of each symptom was graded on a scale ranging from 0 to 3 (0: none; 1: sometimes; 2: daily; 3: each meal). The maximum total score was 15 points for each patient. Total symptom improvement was assessed by comparing the pre- and post-myotomy symptom scores.

 Esophageal manometry was performed by a gastroenterologist before and two months after the treatment of Heller myotomy in all patients. The degree of improvements in symptoms was recorded after the treatment of Heller myotomy in patients. This method was performed by 4-cannula water perfusion system (Medtronic Co, Polygraph HR, USA). The pressure sensors of a polyvinyl catheter (4.5 mm diameter) were placed 5 cm apart. The catheter was passed through the nostrils down to the esophagus, under local anesthesia. Intra luminal pressures were recorded by graph for windows version 2.04 function testing software on a polygraph. The LES pressure was studied by station pull-through technique. Relaxation of the LES was carried out by asking the patient to drink a sip of water. The distance between the LES and sensors was 5 cm. Incomplete or loss of relaxation of LES and simultaneous contraction confirmed the diagnosis of achalasia. 

**Statically analyses**

Patients’ demographic, clinical features and response to treatment were analyzed by SPSS software (version 20, Chicago, IL, USA). The paired t-test was used to compare the criteria before and after treatment. Two-sided p-values less than 0.05 were considered significant. 

**Table 1:** Pre-and postoperative results of functional state of the esophagus assessment

	Pre-Treatment Mean ±SD, (N=20)	Post-Treatment Mean ±SD, (N=20)	P value
LES Resting Pressure (mm Hg)	35.2±1.8	12.7±1.5	0.0001
LES Residual Pressure (mm Hg)	27.7±2.5	13.4±2.4	0.0001
IRP (mm Hg)	25.7±2.9	7.9±1.1	0.0001
Free drinking esophagus (mm Hg)	42.1±3.2	21.9±2.9	0.0001
length of the esophagus (cm)	23.5±0.5	21.6±0.4	0.004
PIP (mm Hg)	45.6±0.6	41.8±1.3	0.002
dysphasia score (unit)	10.5±0.2	3.2±0.3	0.0001

## Results

Twenty patients with mean age ±SD of 30±3.5 years, including 12 males and 8 females were included in this study.

Results of this study showed that, all the diagnostic criteria that were measured before the treatment was significantly different from after the treatment (P<0.05). The average decline in LES Resting Pressure, LES Residual Pressure, Free drinking esophagus, PIP, and IRP were 23.2 mmHg, 14.3 mmHg, 20.2 mmHg, 3.4 mmHg, and 17.8 mmHg, respectively. Also the average decline in the length of the esophagus was1.8 cm and dysphasia score was 7.25 units (Table 1).

Before treatment, one patient was classified as type I (5%), 17 as type II (85% patients), 1 as type I+II (5%) and 1 as type II + III (5%). After treatment (follow-up period of 2 months), 1 patient (5%) had achalasia type I, 10 (50%) had achalasia type II, 6 (30%) had achalasia type I+II and 3 patients (15%) had achalasia type II + III. Results of this study showed that, all the diagnostic criteria that were measured before the treatments were significantly different from those of after the treatment.

Also, we found that type II achalasia is the most common subtype of achalasia and patients with type II achalasia responded better to Heller myotomy compared to the others. 

## Discussion

Since the etiology of esophageal achalasia is not recognized, the management of choice for achalasia remains controversial. 

Pharmacotherapies are not valuable in the long term and botulinum toxin therapy provides suggestive short-term help for achalasia patients (19, 20). The choice of pneumatic dilatation is frequently based on the fear of the operation, its lasting benefits surpass all other nonsurgical treatments, and it is cheaper than surgical myotomy (21-24). However, many retrospective studies and prospective randomized trials have shown that the results obtained by myotomy more effectively than those obtained by pneumatic dilatation (25-27). The development of myotomy has a high success rate with minimal morbidity; therefore further patients are being referred for myotomy as a primary treatment (21-23). 

Results of this study showed that, all the diagnostic criteria measured before the treatments were significantly different from after the treatment. Our findings are similar to those of Shiino et al. which have reported that, persistent dysphagia and postoperative gastroesophageal reflux are the most cited reasons for surgical failure but dysphagia relief is maintained in 85–100% of Heller myotomy patients (21). Lyass et al. have shown that Heller myotomy is highly effective in relieving dysphagia in patients with achalasia (28). In addition, Aghajanzadeh et al. reported that short-segment cardiomyotomy decreases the LES gradient and relieves obstructive symptoms (29). These results show that myotomy, can be used for treatment of the patients with achalasia and manometry can be used for assessment of efficacy for patients with achalasia who were treated by myotomy.

In conclusion, Heller myotomy is highly effective in relieving dysphasia among Iranian patients with achalasia. Results of this study show that type II achalasia is the most common subtype of achalasia and patients with type II achalasia had a better response to Heller myotomy compared to others. Based on the new classification of achalasia and similar to our result, previous studies showed that type II is the most frequent subtype of achalasia (17). These results suggest that each subtype could be related to diverse disorder mechanisms and that subtyping might play a significant role in forecasting the treatment reaction (2,16,30-34). Therefore, the subtyping of achalasia by manometry may authority the clinician to direct therapy and predict the outcomes (18).
